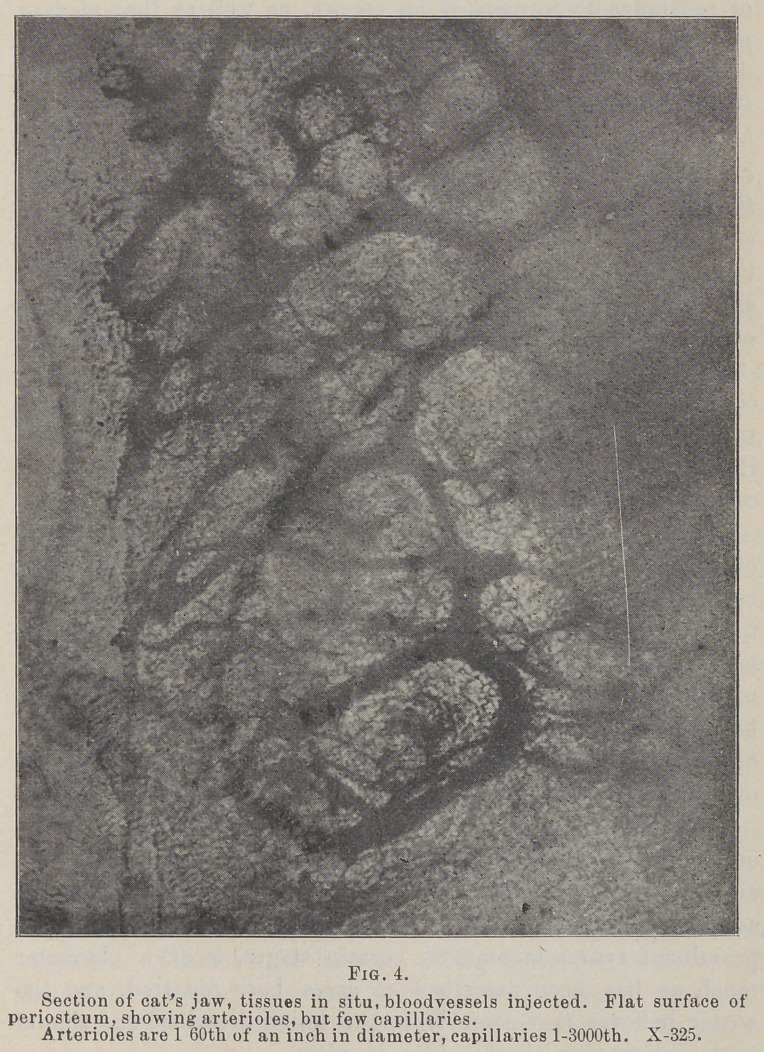# Periosteal Caries from Bacterial Origin

**Published:** 1899-08

**Authors:** M. H. Fletcher

**Affiliations:** Cincinnati, O.


### THE DENTAL REGISTER.

Vol. LIII.] AUGUST, 1899. [No. 8.

### Communications.

### Periosteal Caries from Bacterial Origin.

BY M H. FLETCHER, D.D.S., M.D., M.S., CINCINNATI, O.

Abstract of paper read before the Section of Stomatology, Amer. Med. Ass'n, Columbus, June, 1899.

Of recent years my attention has been called to a number of cases of neuralgia, the exciting causes of which were obscure, as is frequently the case.

Amongst others was a case of supposed tic doloureaux, accompanied by the apparent death of the periosteum over a larger portion of the lingual surface of the inferior maxilla of left side. While the exciting cause in this case may have been central, it apparently was peripheral; it yielded to local treatment, which fact strengthened belief in the peripheral origin. The finding of this lesion was the result of desperate effort, every logical treatment, both systemic and local, having been resorted to, aside from nerve section, and all without relief.

REPORT OF THE CASE.

Mrs. X, age fifty-five, suffered with paroxysmal pains resembling tic doloureaux. These continued with increasing tendency for three or four years, always including the first molar; this was the only tooth remaining back of the first bicuspid, and it was finally extracted, after having been devitalized and properly treated. For a short period this gave some relief, but the paroxysms soon returned with increasing severity.

There was nothing abnormal in the microscopic appearance of the bone, flesh, or mucous membrane, and no tenderness nor swelling, and no pus could be detected. Thinking possibly something abnormal might be discovered with a magnifying glass, one was used, and a tiny slit was found, not to exceed one-sixteenth of an inch in length and situated on the summit of the alveolar ridge at the former seat of the first molar.

My first impression was that I should find here a portion of the root of the tooth, but none could be discovered. The probe, however, after being forced between the lips of the opening, went into a large pocket on the lingual surface of the bone without further resistance. Careful probing showed this pocket to reach from the summit of the alveolar ridge to the inferior border of the jaw, and from the first bicuspid back to the angle, including the inferior dental foramen. The surface of the bone, instead of being rough and necrotic, had the feel of being eburnated and without periosteum. Of course the exact conditions of such cases can only be verified by post-mortem examination, and as yet no opportunity has offered for such investigation.

It is held by others that the periosteum in this case is probably still present, and either divided or entirely adherent to the supervening soft tissues, and I believe that all known laws of pathology would support the hypothesis that the periosteum is dead in this and all similar cases.

In studying the subject with the conditions in mind as recited in the above case, many old cases may be recalled and many new ones have been presented which are interesting as bearing on the pathology of the subject.

Two or three will be described as typical, and, I believe, will show sufficient reason for advancing the hypothesis of necrotic or carious periosteum and peridental membrane. The writer holds that the periostum and peridental membrane are identical in everything excepting thickness.

CASE TWO.

Miss Y, age thirty-five, after having suffered with neuralgic pains on both sides of the face for some months, presented herself for treatment. The pain was paroxysmal in character, distributed on both sides of the face, being excited by foods, especially sweets, sours and salts, sometimes lasting for hours, there being very few meals passed without more or less pain, the paroxysms becoming more frequent and severe as time passed.

Fig. 1.

Section of cat's jaw, tissues in situ. Bloodvessels injected. *B*, Bone; *A.i.P.* Arteriole in Periosteum; *D*, Dentine; *B. i. Pl.*, Bloodvessels in Pulp; *C. i. P.* Capillaries in surface of Pulp. X-300—

The greater intensity and more frequent location of pain was that of the lower molars on the left side.

On examination little or no calcareous deposit was found, excepting on the backs of the lower centrals, but the pain never seemed to locate itself in this place.

About the molars and bicuspids, more especially around the molars on the left side, there was very apparent difficulty in the form of necrosed bone. The septa, especially between all the molars, were more or less dead, some more than half way to the apices from the necks of the teeth. About the latter mentioned teeth in some places a smooth broach could be pushed clear to the apices of the roots, without pain or bleeding, showing the periosteum to be absent in much of the socket; but on cutting the septa away a sensitive and bleeding condition was reached, about half way from the neck of the apex. The lingual and labial portions of the bone did not present so extended a lesion, but death of the periosteum seemed to progress more rapidly and covered a much greater extent of surface than the dead bone.

In none of these localities, where there was little or no tartar, was there much apparent inflammation in the soft tissues, and no tenderness in the bone and flesh, until live tissues were reached below the necrosis. Where the calcareous deposit was greatest (on the backs of the lower centrals), there was some redness and possibly some pus with the bleeding, but no yellow or watery pus, as in cases of typical pyorrhea.

CASE THREE.

Mr. Z, age fifty, indicated constant pain, varying in intensity, sometimes in angle of jaw and ear, but often in the lower first molar on the left side, or in vicinity (the only molar remaining on that side below). Half of the crown of this tooth had been worn away and a protection of gold had been built on, and a large cavity in the anterior approximal surface had also been filled with gold. This gold-work was going to pieces, and had to be replaced. It was found that decay had proceeded under the approximal filling, so that the pulp had to be capped, and after this the pain increased in intensity and frequency of paroxysms. The next step was to remove the pulp and obliterate the pulp cavity and root canals. This gave only slight improvement, so search was made for bone and periosteal trouble. It was discovered that a probe could be passed underneath the soft tissues on top of the ridge for fully half an inch behind the first molar, the same condition of bone surface being present here that was found in Case 1 (Mrs. X). Farther search also showed the septum to be largely dead between the molar and bicuspid.

Numerous cases could be reported to fill in all the varieties of this trouble between the two extremes, both in extent of lesion and pain, and this not only from my own records but from those of some of my confreres, notably Drs. Heise, Callahan and

Fig. 2.

Section of cat's jaw, tissues in situ, bloodvessels injected. *B. i. pl.*, Bloodvessels in Pulp; *D*, Dentine; *P. C.*, Pericementum; *P*, Periosteum; *B. i. P.*, Bloodvessels in Preiosteum; *B*, Bone. X-300+

LeFevere, who have become interested in this particular lesion as distinct from pyorrhea alveolaris and other lesions of bone.

The principles of treatment have been the same in all this class of cases, viz: Removal of dead bone, if such could be discovered, and the sterilization of the pockets with escharotics. The remedies usually used have been 5 percent alcohol as a menstruum for tincture of iodine, the strength of the latter used, according to locality and symptoms, from almost full strength to a 5 percent solution, and to this was added 2 or 3 percent of oil of cinnamon.

In all cases recovery has been slow, varying from a few weeks to a year in duration.

In going back over these cases as to the extent of lesion, we have, first, cases in which the septa between the teeth become denuded of periosteum; then follows death of the septum, the former lesion at times invading the alveolar process on the lingual or buccal surface of the bone, although the soft tissue, aside from the septa of gum, still remain in shape and show little signs of inflammation.

Now, as the trouble creeps down on the outside or inside of the jaw, as in Case 1 fully described, where the bone is much thicker and the collateral sensation much more extensive, the surface of the bone feels to the touch of an instrument as though it was eburnated and not dead; and, if the sense of touch can be relied upon, the periosteum is absent as far as the surface of the bone is involved; it may be adherent to the supervening soft tissues, but the writer is of the opinion that it has been destroyed.

If progressive lesion of the periosteum on the outer or inner surfaces of the alveolar process is accompanied with confinement of pus, as it often is, we then have what is recognized as gingival abcess, and if the lesion about the roots and peridental membrane is accompanied with a perceptible flow of pus it would be termed pyorrhea alveolaris; but both of these conditions are to be distinguished from the one under discussion. Nevertheless, this lesion may result in a collection of pus, and if this occurs a name is given it according to its locality, such as felon (onchia) empyema of the maxillary sinus, etc. Another phase of this lesion, in the writer's opinion, is that this peridental caries or mycotic destruction *precedes the so-called uric acid or serumal deposits about the roots of the teeth, which deposits are secondary to a lesion and not primary to it.*

There would seem to be no impossibility in the position that

FIG. 3.

Section of cat's jaw, tissues in situ, bloodvessels injected. *B*, Bone; *P.D.M.*, Peridental membrane; *D*, Dentine; *B. i. Pl.*, Bloodvessels in Pulp, showing network of capillaries on the surface. X-300—

the periosteum may be destroyed and the bone remain alive, as in Case 1, since the collateral circulation could certainly supply the necessities of life for an indefinite period to a bone as large as the maxilla. Then, too, the progress of the disease is so slow that the surrounding tissues have ample time to adjust themselves to the new conditions, and we all know that nature is ever ready to adapt herself to change when compelled to in order to preserve the life of the organisms.

As to the periosteum being lifted off the bone and remaining adherent to the supervening soft tissues, this can only be decided by post-mortem examination.

I believe it will be admitted that all lesions of the periosteum are attended with pain of a neuralgic character. In cases of confined pus within, or upon the surface of the bone, the pain is often almost beyond endurance.

In osteomyelitis previous to the death of the parts, Senn says: "Pain may be absent at the seat of the necrosis, and referred to some other part or locality." This is true in the cases observed, for the sensation was largely referred to some other place or locality rather than the seat of the lesion, many times the patient not being able to locate it.

ETIOLOGY.

As to etiology, Senn further remarks, under necrosis, "The same bacteria which produces inflammation frequently, if present in sufficient quantities, also causes cell necrosis," and he quotes his authority for such belief. The above statement and the supporting authority, in company with the knowledge of the methods and destructive attacks of bacteria on dentine, could almost convince one that, once a suitable culture of bacteria or cocci had been started in the horn-like fibers of periosteum, *that they could produce continuous destruction of the membrane, much as they do that of dentine*, and this opinion is held for the following reason:

The study of the periosteum in my own laboratory shows it to be a very compact membrane of connective tissue fibres, and histiologically much more the nature of the hard tissues than of the soft, hence the application of the word "caries." In one of the layers it is found carrying arterioles in great numbers, *but*

*having very few if any capillaries anywhere in its substance,* and no blood-vessels at all in one layer, although the supervening soft tissues are plentifully supplied with them. (See accompanying photos.)

If this is true, then it seems rational to hold that this mem-

FIG. 4.

Section of cat's jaw, tissues in situ, bloodvessels injected. Flat surface of periosteum, showing arterioles, but few capillaries.

Arterioles are 1 60th of an inch in diameter, capillaries 1-3000th. X-325.

brane during life can and does form a good and sufficient pabulum for the growth and development of bacteria, and this to its own destruction, the neighboring capillaries not being in a position to combat the inroads of the enemy much better than they would in caries of dentine. In capillaries lie the power of resistance to inroads of an enemy, as well as that of recuperation. It being known that migration of leucocytes and diapedesis takes place almost exclusively in the capillaries, protection and repair being among their most important offices, *hence in tissues where they are absent or few, bacteria might be expected to thrive, and the writer is of the opinion that they do and that the lesion in question is resultant upon the conspicuous absence of capillaries in the periosteum*, and they seem especially few in the periodental membrane, as the accompanying photomicographs show, which fact may explain many of the phases of the diseases of this membrane and the alveolar process.

The exceedingly slow recovery of these cases under germicidal and stimulating treatment would also tend to support the hypothesis of the death of the periosteum by bacteria; and, if the death is caused in this way, then the process might be called caries of periosteum, or periosteal caries.

This hypothesis, however, can not even attain to the dignity of a theory until some one with ample time and opportunity takes up the subject and carries it through clinical and laboratory tests sufficient to establish it beyond question. The writer is convinced, however, that one would be highly justified in undertaking such a series of tests; and, if such a theory could be established, many cases of so-called rheumatism, obscure neuralgia and headaches could be traced to a progressive lesion or death of the periosteum in the bones involved.

In what seems to be complete recovery from amputation, or compound fracture, pain is often continuous and severe, and not easily accounted for, and in chronic diseases of the accessory air cavities and the mastoid cells neuralgic pain is a prominent symptom and, at certain stages, is of a character much as has been described in cases here reported, and the writer believes may come from the lesion described and should be investigated with that idea in view.

Regarding periosteal caries from bacterial origin in cases of amputation or fracture, and where thoroughly aseptic methods have been employed, one might be skeptical; but the infection may be carried through the blood from many distant sources, and if there were no other source the gums, teeth, and alveolar process are in many people the most perfect of incubators and are abundantly supplied with pathogenic microbes, especially that of pus, so that from this source, if from no other, pus microbes and possibly other pathogenic germs could be taken up by the blood, and by locating at the point of least resistance (locus minoris resistentiæ) result in the lesion under discussion, it being known that under different environment the same microbe may produce different results.

Roswell Park, under "Source of Infection," says:

"The oral cavity and pharynx are never free from bacteria. Miller has studied over one hundred species that he has found under various circumstances in the human mouth. Some of these are pathogenic, others are apparently absolutely innocent. Many of the forms which grow in saliva will not grow in ordinary media. Miller has also shown that all forms of dental caries are but expressions of bacterial invasion, even of those apparently most solid structures, the teeth; and of late we have been taught more fully that such invasion may extend far beyond the confines of the teeth alone and may spread to various, even distinct parts, and produce possibly fatal mischief. Abcesses in the brain and extensive septic infections have been clearly traced to invasion along the line of the dental troubles. One of the most virulent of all the common inhabitants of the mouth is the pneumococcus of Frankel, which, passing into the general circulation through the tonsils or other possible ports of entry about the mouth, cause serious septic and inflammatory disturbances in widely distinct regions. Aside from dental caries, a widely-opened port of entry is often afforded by those ulcerations around the margins of the gums which are produced by accumulations of tartar. Disease in the antrum of Highmore, for instance, and many other local destructions, are frequently caused in this way."

Now, is it not rational to believe that in lesions of the periosteum, whether traumatic or otherwise and regardless of what bone of the body it may be upon, that we could have the progressive destruction of that membrane as above described ?

The writer believes we can, for we certainly have in a very large percent of people the constant source of infection, and in every person the conditions in the minute anatomy to permit of what might be termed periosteal caries from bacterial origin.

---

[In the discussion of this paper the term "caries" was largely objected to, and that of "ulceration" and "necrobiosis" suggested; "ulceration" is not appropriate (in the writer's opinion), *for it implies death of tissues in which the cells are composed of liquid protoplasm*, a condition which does not obtain in the cells of the periosteum. The same is true of "necrobiosis," or "coagulation necrosis," which processes attack the cells with protoplasm, and not the connective tissues between the cells, *the periosteum, being composed of connective tissue, could not come under any of these heads*.

Klebs found that "karaolysis is due to the action of chemical products of bacilli," hence "mycotic necrosis," or "karaolysis of periosteum," seem next appropriate to "caries."

Miller, in treating of dental caries, says: "Dentine may be defined as a dense, glue-giving basis substance, etc. The relations of Sharpie's fibers to the progress of decay in the cementum is very significant, etc. They (the Sharpie fibers) thereby facilitate the invasion of bacteria, etc." Now Sharpie's fibers, when decalcified, are composed of connective tissue substances, and when in situ are often continuous with the fibers of the periosteum, being like it in composition.

Now, all the connective tissue substances of the body are glue-giving, and the substance of the periosteum is composed entirely of these fibers; hence "caries," which is always mycotic, seems as appropriate for the bacterial destruction of periosteum as to the other glue-giving tissues of the body. "Mycotic periosteal caries" might possibly be better than the title used, but "caries" signifies mycosis, hence does not need qualification.]

## Figures and Tables

**Fig. 1. f1:**
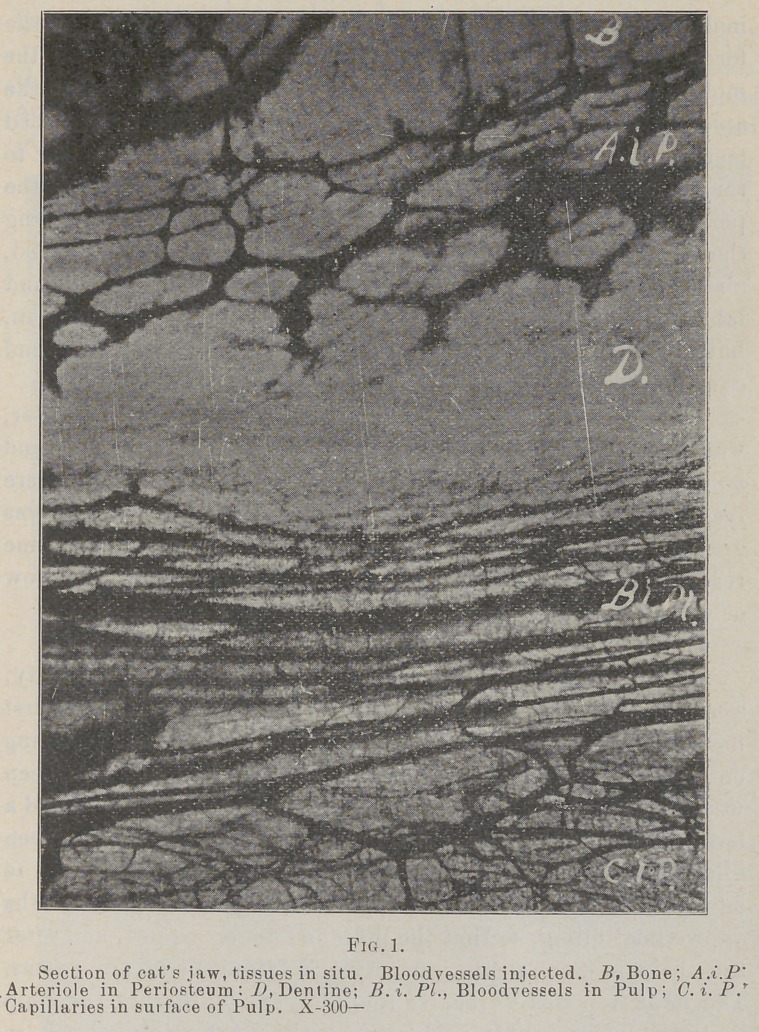


**Fig. 2. f2:**
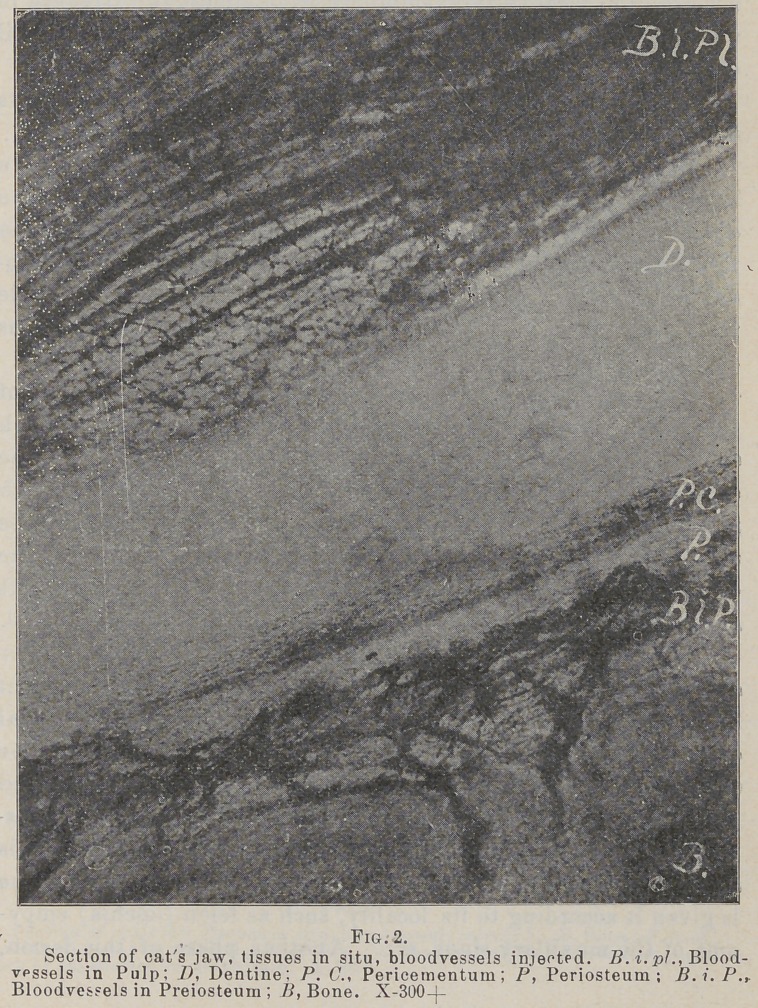


**Fig. 3. f3:**
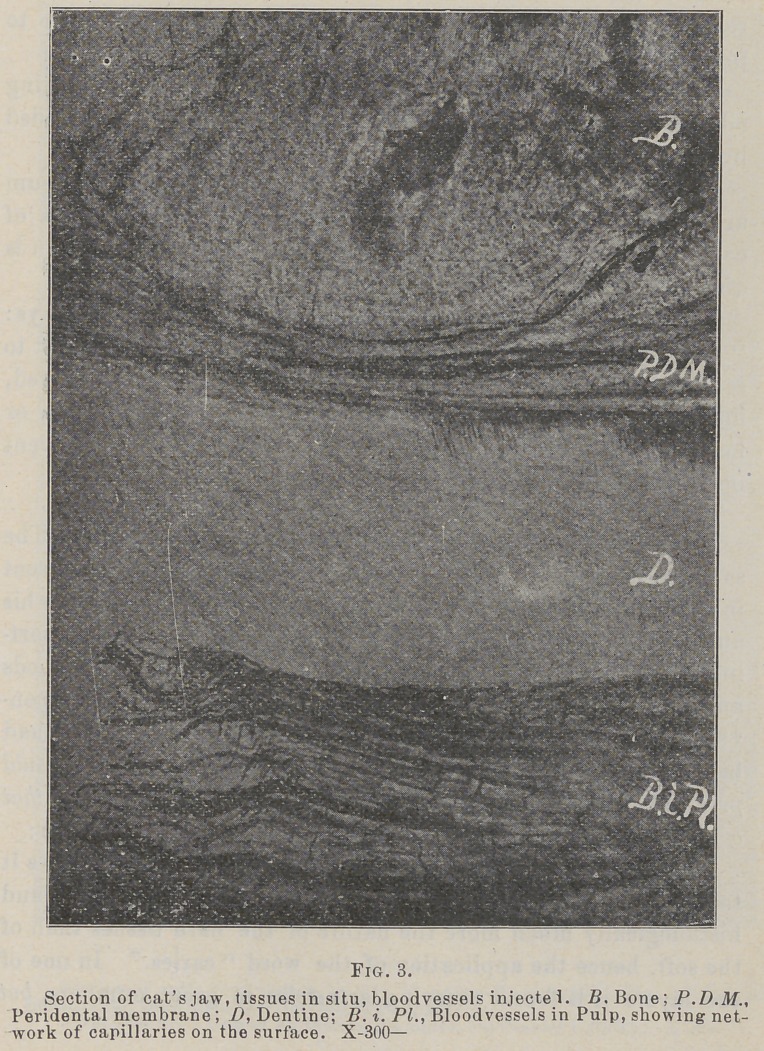


**Fig. 4. f4:**